# Necrosis of Inferior Maxilla from the Vapor of Phosphorus. Removal of the Entire Lower Jaw—Recovery—Remarks upon Phosphorus Disease

**Published:** 1856-07

**Authors:** James R. Wood

**Affiliations:** Surgeon to Bellevue Hospital, New York, etc., etc.


### ARTICLE IX.

*Necrosis of Inferior Maxilla from the Vapor of Phosphorus. Removal of the entire Lower Jaw—Recovery—Remarks upon Phosphorus Disease.* By JAMES R. WOOD, M. D., Surgeon to Bellevue Hospital, New York, etc., etc. With Illustrations.

*Case.*—Cornelia S., born in Germany, aged 16; admitted into Bellevue Hospital, December 17, 1855. She came to this country at the age of three months; eight and a half years ago her father died of phthisis; four years ago her mother died of fever. She has enjoyed good health up to the time of her present trouble. Two and a half years ago she commenced to work in a match factory on Second Avenue, in this city, where she remained six or seven months. She then left this factory and entered another on Norfolk street, where ventilation was very imperfect. Her business was "packing," the "dipping" being done in another apartment. She continued at her occupation, working eight hours a day, and feeling perfectly well, until about the 1st of May, 1855. At that time she was seized with toothache, and swelling on the right side of the lower jaw. To relieve it, her gums were lanced, and, finally, the tooth extracted. After this the pain ceased; but the swelling gradually increased, until a spontaneous opening formed on the under side of the jaw, with a discharge of pus, which has continued since. She remained in the factory until one week previous to her admission into the hospital.

Upon examination after her admission, the inferior maxilla was found necrosed on the right side and partially on the left. Her general health was good. Her jaw was painful, and that side of the face swollen. The discharge was at times profuse, and a part of it took place through the buccal cavity, rendering it very annoying. Her appetite was good, but mastication difficult and painful. She never had had syphilis. The necrosis gradually extended, but her general condition remained good.

On the 19th of January, 1856, thirty-three days after her admission, I proceeded to remove a portion of the necrosed bone upon the right side, intending to leave both the symphysis to which the lingual muscles are attached, and the ramus of the jaw. No anæsthetic was used. The patient was placed on the operating table, with her head and shoulders elevated, and her face turned towards the left side. The external incision commenced midway between the angle and condyle of the right side, and extended along and under the base of the jaw, terminating one quarter of an inch below the symphysis menti. The soft parts were next divided, and the periosteum carefully separated from the bone. A chain saw was then passed under the jaw into the mouth, half an inch to the right of the symphysis, and the bone sawn through. The saw was again passed under the jaw, at its angle, for the purpose of dividing the bone at this point, but, unfortunately, on attempting to work it, the chain broke. I now seized the bone at this point with Liston's forceps, and endeavored to divide it, when it was readily discovered in this attempt that the jaw was necrosed to its articulation. I then endeavored, with the forceps, to remove the jaw entire upon the right side, and succeeded, with considerable effort, in completely enucleating it from its periosteal covering.

But little hœmorrhage occurred, and no vessel required the ligature. The parts were brought in apposition with sutures, and adhesive strips and cold water dressings applied.

*January* 20.—Pulse, 90; no pain; slept well last night.

*January* 22.—Wound dressed for the first time; a small part had united by first intention, the remainder in good condition; no pain.

*January* 26.—Wound entirely healed. An old fistula on the right side, still continues to discharge purulent matter.

While the right side had so greatly improved and apparently left no remnant of the former trouble, the disease was extending upon the left side, involving new portions of the jaw, and giving rise to an immense secretion of intolerably offensive pus. It was, therefore, deemed advisable to attempt the removal of the remaining diseased mass. Accordingly, on the 16th of February, twenty-eight days after the first operation, I removed the remainder of the jaw. The whole of the opposite side I thought dead or dying. At the symphysis it had almost separated itself from the soft tissues, leaving only slight attachments for the lingual muscles. In removing this side of the jaw I designed to leave that part of the symphysis to which these muscles are attached, partly to avoid the liability of the patient's tongue receding into the larynx, but principally to leave an isthmus which should preserve the contour of the chin, and serve as a point of departure for new bone, which would form the periosteum, thus far carefully preserved.

The external incision was similar to that of the opposite side, except that it terminated one-half an inch below and to the left of the symphysis, leaving half an inch of healthy tissue between it and the other cicatrix. The soft parts were next divided, and, with the periosteum was dissected from the bone, both on its external and internal surfaces, as in the previous operation. An assistant now took hold of the symphysis, and a chain saw was passed under the jaw into the mouth, from half to three-fourths of an inch to the left of the symphysis. My object in sawing through the bone to the left of the mesial line, was to prevent the accident previously mentioned; but, unexpectedly, the moment the bone was divided, the central portion left at the chin escaped from its attachments, by simple enucleation, into the hand of the assistant, and the tongue was immediately swallowed. Respiration instantly ceased, and suffocation impended; but, with a pair of strong forceps, the tongue was seized and replaced, and a ligature passed through it, and secured externally. It was now ascertained that that portion of bone above the angle, was not necrosed, as on the opposite side; but it was decided that the disease could not be arrested, without its entire removal. To complete the operation, the soft parts were separated from the ramus in conjunction with the periosteum, the capsular ligament was opened anteriorly, and a chisel passed over and behind the condyloid process, and by this means the bone was disarticulated. Not a single vessel was tied. The wound was dressed with sutures and adhesive strips. Twenty drops of laudanum were ordered, to procure sleep.

*February* 17.—Pulse, 112; slept well; wound glued together throughout its whole extent; considerable swelling, but no redness or increase in temperature. Left eyelid œdematous and closed. Wound re-dressed with adhesive strips, and lotio plumbi et opii applied.

*Feb.* 18.—Face much swollen; some pain over region of the jaw; pulse, 138, and irritable; wound united more firmly, except about half an inch near an old fistulous opening, which discharges pus and saliva. Four ounces of wine ordered to be given during the day—and the lead and opium wash continued.

*Feb.* 19.—Pulse, 100; pain and swelling greatly diminished. Left eye partially open—continue treatment.

*Feb.* 20.—Pulse, 98; no pain; some œdema of palpebræ. Eye easily opened; wound united by firm adhesions throughout its whole extent; no fistulous openings on left side of the face. Appetite good; diet consists of soups and farinaceous substances; unable to masticate solid food—continue the lead and opium wash.

*Feb.* 21.—Swelling of face nearly subsided; eye open; ligature in tongue removed.

*Feb.* 23.—Swelling entirely subsided. The contour of the face is perfect. All the movements of the tongue, and those pertaining to the jaw, are preserved—such as protrusion of the tongue, lateral motion, deglutition, etc.

From this time until the 4th of March, the patient did well, and every thing seemed to favor a permanent and radical cure. On the 4th, she went out on a visit to her friends. She was thinly clad, and suffered from the cold. The next day, March 5, the left side of her face was swollen, hot and painful. She had some thirst, a light fur on the tongue, and an accelerated pulse—ordered a cathartic, with lead and opium wash.

*March* 6.—Patient feels much better; all inflammatory symptoms have subsided. Two fistulæ have formed in the track of the cicatrix, which are discharging healthy pus—ordered a light flaxseed poultice.

*March* 12.—Two small pieces of bone discharged through the fistulous openings.

*March* 20.—Fistulæ entirely closed.

During the progress of the case no unfavorable symptoms appeared. The incisions healed with remarkable rapidity. The patient had a good appetite during the whole time. The contour of the face is preserved with remarkable accuracy. The cicatrices are entirely concealed from a front view, and all the motions pertaining to the jaw and tongue are unimpaired. New bone began early to form, and small pieces have already separated.

The illustrations exhibit, accurately and beautifully, the appearance of the inferior maxilla, when the different portions of the bone were properly united, and also the amount of deformity which remains after the removal of such an integral portion of the skeleton frame work of the face.

I take this occasion to acknowledge my indebtedness to Dr. Geo. Amerman, house surgeon to Bellevue Hospital, for his attention to my patient, and the foregoing details of the case.

*Remarks.*—Phosphorus disease, or necrosis from exposure to the fumes of phosphorus in the manufacture of lucifer-matches, was first noticed in Germany. Lorinser, of Vienna, published the first account of this disease in 1845, and reported a number of cases. Soon after, Heyfelder, of Erlangen, and Strohl, of Strasburg, published cases; and in 1847, Drs. Von Bibra and Geist,* published a separate work. In the following year, accounts of the disease were published in England; and in noticing a case, in the surgical reports of Guy's Hospital (1846–47) of separation and exfoliation of the lower jaw, from exposure to phosphorus, in the manufacture of lucifer-matches it is stated that the disease was previously noticed to be not uncommon in those working in phosphorus. Mr. Stanley alludes to this disease in his Treatise on Diseases of Bones. Cases have been occasionally reported in English periodicals; and in the Lancet for 1850, (vol. i, p. 41,) there is an interesting clinical lecture, by Mr. Simon, on this subject, with the full details of a case. Phosphorus disease does not seem to have been frequently noticed in this country, if we may judge by reported cases; yet the causes exist among us in all their intensity. I am aware, indeed, of but a single case which has been placed on record, and that was observed by Dr. Bigelow, of Boston. That this disease is more prevalent in this country, than might be inferred from this single case, is evident from the several cases appended to this paper, which I have been able to collect, and the case kindly communicated by Dr. Van Buren.

As this affection has not been brought before the American reader in any detail, the following summary of what is known of its nature, progress, and results, may not be inappropriate in this connection:

That phosphorus is the destructive agent in this disease, has been proved by experiments upon animals. Rabbits exposed to the fumes of phosphorus, under circumstances similar to those which determine the disease in man, are

* *The Diseases of the Workmen employed in Lucifer-match Manufactories, and especially the Affection of the Maxillæ, produced by the vapors of Phosphorus, etc.* By F. Ernst Von Bibra, Ph.D., and Lorenz Geist, M.D., Erlangen, 1847. See also *British and Foreign Med. Chir. Review,* 1848, vol. i, p. 446.

similarly affected. Another fact seems clearly established, viz: the vapor of phosphorus must come into immediate contact with the periosteum or bone, in order to excite the morbid process. This explains, in the first place, why but few, comparatively, are affected who work in these manufactories; and, in the sccond place, why the lower jaw is more frequently the seat of the disease than any other bone. For it appears that those only suffer who have decayed teeth—the defect in the teeth allowing the fumes of phosphorus to penetrate to the periosteum. So important is this latter fact, that the government of Erfurt has passed a decree, that no person having decayed teeth shall be allowed to work in lucifer-match factories. In a factory in this city, no workman is allowed to return to his work for a week, after the extraction of a tooth.

That particular part of the work which gives rise to the greatest quantity of vapor of phosphorus is the most dangerous to operatives. This occurs in the process of preparing the paste, and in dipping. In the first process, a high degree of heat is necessary, and large quantities of the fumes of phosphorus are given off, which fill the rooms. In the second, the paste is spread upon a metal plate, with a temperature sufficiently high to keep it liquid, over which the dipper stands, and necessarily inhales the vapor which arises. Where the ventilation of the establishment is well conducted, the "dipper" is the only operative affected by the phosphorus; but where the ventilation is bad, and the fumes of the phosphorus, disengaged, not only during the process of mixing and dipping, but also in counting and packing, are confined, workmen engaged in other departments are similarly affected. This fact finds striking confirmation in the history of lucifer-match factories of this city. In the old factory in Twelfth-street, the ventilation was poor, and the mixing room was in communication with the work room. As a consequence, whenever the paste was prepared, the whole room became filled with the suffocating vapor of phosphorus. In this establishment, phosphorus disease seems to have been not uncommon. In the new factory, the phosphorus room is in a separate building; and so perfect is the ventilation, that there is scarcely a smell of phosphorus in the building. No case has yet occurred in the new factory.

The general effects of phosphorus upon the workmen in these factories, are differently stated by different writers. The German authors do not seem to refer the diseases of operatives to this cause; but, on the contrary, regard the laborers in these establishments, as healthy as those in any other. French writers, however, ascribe to the inhalation of the fumes of phosphorus, certain bronchitic affections under which this class of persons are found to labor. English observers agree with the German, in regarding phosphorus vapor as harmless to the individual; and some even allege that the operatives in these factories, enjoy better health than before entering them. I have not been able to learn that the workmen, in these factories in this city, suffer unusually from bronchitis, or indeed any other affection which could be traceable to phosphorus, except the disease under consideration. Two intelligent medical students from my office, Messrs. Bird and Johnson, have visited the lucifer-match establishments of New York, and have been kindly received by the proprietors, who gave them every opportunity to thoroughly examine the premises. In their report to me, with the appended cases which they were able to collect, the following note is made of the appearances of the operatives :—"They seemed as healthy as those of our cotton factories in Lowell, or our woolen factories in Lawrence, or our flax factories in Andover, Mass."

The peculiar form of disease here considered, is a periostitis. It has been a question,—whether the disease is excited by direct contact of the phosphorus with the periosteum, or whether it does not first enter the blood, contaminate the system, and secondarily induce necrosis. This question would seem to be definitively settled by the following considerations : 1.—Operatives exposed to the fumes of phosphorus do not suffer from any special or general malady, showing contamination of system, or the existence of a cachexia. 2.—The disease attacks only denuded bones. So well established is the fact, the operative is considered safe until he has carious or extracted teeth.

We consider it established then, that the phosphorus must find access to the periosteum, when the morbid process is set up. It more often affects the maxillary than other bones, for this reason ; and the inferior maxilla than the superior. That other bones are equally affected, when the phosphorus vapor reaches the periosteum, is proved by experiments upon animals.

The frequency with which the different bones of the face are affected in this disease, is exhibited by the following collection of cases :—

| Whole No. | Max. Sup. | Max. Inf. | Max. Sup. and Inf. |
|---|---|---|---|
| 66. | 22. | 36. | 8. |

The pain of the jaw, which ushers in the disease, is generally mistaken for toothache. It is usually slight at first, and intermittent, and is due to the slow process of periosteal inflammation which results in the formation of a lamina of bone beneath the periosteum, and around the old bone. This takes place around the base of the jaw, owing to the gravitation of the exudation from the inflamed periosteum. This, the first stage, is chronic and may be indefinitely prolonged, without causing much inconvenience to the patient. The second stage begins with an attack of acute inflammation in the diseased part, excited by cold, or otherwise; there is great pain and swelling of the soft parts; the new formation is destroyed, and discharged, with an abundance of offensive pus; and the old bone remains a sequestrum in the midst of the products of suppuration, to be subsequently discharged in successive portions. This stage is attended with great suffering and constitutional disturbance; and not unfrequently patients die from exhaustion during this process of suppuration, or from gangrene of the soft parts. If the disease pass on unarrested, the jaw becomes more and more involved, large portions exfoliate, and the whole finally becomes implicated. Few survive to this period, and a still less number witness the completion of the morbid process, in the discharge of the entire jaw. Mr. Stanley exhibited a patient of St. Bartholomew's Hospital, suffering from this disease, whose entire lower jaw had exfoliated, excepting one condyle.

The prognosis in these cases is very unfavorable. When the disease first comes under notice, the periosteal inflammation has generally long existed, and new formations already separate the bone from its covering. More frequently the suppuration is established, exfoliations of bone are taking place, and the whole morbid process is in active progress. The system now breaks down under the exhausting discharges and poisonous emanations from the jaw; and the miserable subject of this destructive disease falls a victim to its inroads upon his strength, long before the completion of the process of exfoliation.

The regeneration of bone, in cases where extensive necrosis of the jaw occurs, or where it is entirely removed, as in the present instance, is an interesting and practical question. From the investigations of Von Bibra and Geist, we learn that the new deposit derives its nutrition from the periosteum only, and is, therefore, the product of this membrane. Unlike callus, it has no communication of the Haversian canals with the bone upon which it lies, while its medullary canals are vertical to those of the bone. They conclude that the new formation has a lower degree of development than true bone. The following is the average of several analyses of bone and the deposit, made by these authors; and, considering the authority of Von Bibra in the chemical examination of bone, they are worthy of note:—

| Bone. | | | Deposit. | | |
|---|---|---|---|---|---|
| Organic constituents | | 31.42 | Organic constituents | | 38.16 |
| Inorganic | " | 68.58 | Inorganic | " | 61.84 |
| | | 100.00 | | | 100.00 |

The excess of organic matter in the deposit is striking, and it would be interesting to know in what relation this deposit stands to the new bone. Some authors doubt the possibility of new bone being formed in these cases; but the case under consideration proves their reasoning untrue. Although there may not be a complete regeneration of bone, the reproduction has evidently begun, and small portions have already separated. As the periosteum, for the most part, still remains, there seems no reason why new bone should not be formed; unless the peculiarity of the periosteal inflammation excited by the phosphorus prevents it. The fact just stated, that bone, or a substance strikingly resembling it, already exists in the track of the bone removed, refutes the supposition.

The treatment of this affection in the early stage is that adapted to periostitis, and in the later stage, necrosis. Free incisions of the gums, both to relieve the tension which results from inflammation of the periosteum and to procure local depletion, will be required. These incisions should be made wherever there is inflammatory swelling, and freely down to the bone. General antiphlogistic remedies will be useful, according to the condition of the patient. When suppuration is established, tonics should be freely administered, to sustain the general health, and exercise in the open air enjoined; locally, detergents may be used with benefit; such as, gargles containing astringents,—myrrh, or chlorides, as the individual case may demand. These measures, however, are but adjuvants in the process of exfoliation.

In the advanced stages, where necrosis has taken place, and nature is endeavoring to separate the sequestrum, an opposite plan of treatment is indicated. An immense discharge of fetid matter issues from the diseased gums, rendering the patient's life miserable, and disgusting to his attendants; his system gradually gives way, and death almost inevitably closes the scene, unless art comes to the assistance of nature. In this, the last stage of the affection, surgical interference seems imperatively demanded. I am aware that some surgical authorities advise to leave these cases to nature, and simply sustain the system. But if we had not reason and experience in analogous diseases to guide us in this last extremity, we certainly have in the case already detailed a clinical fact worthy of consideration. The benefit which this patient derived from surgical interference was never surpassed in my experience. The first operation was followed by the most decided improvement of her general condition, and the last has restored her to a comparative health. I should, therefore, always advise to remove the dead bone as early as possible, and thus relieve the system of a source of great irritation, which nature labors long and often ineffectually to accomplish. If this is judiciously effected, and the general health preserved, we may confidently anticipate that by a regeneration of the osseous tissue, not only will the deformity be inconsiderable, but the functions of the inferior maxilla will, to a considerable extent, be preserved.

*Case* 2.—(Communicated by Dr. Wm. H. Van Buren.)—James O'Donnell, a native of this city, 24 years of age, was admitted to the N. Y. Hospital on the 21st of February, 1856, with necrosis of the left side of the lower jaw, accompanied by very considerable swelling, hard to the touch, and presenting the shape and general physiognomy characteristic of necrosis of the lateral portions of the inferior maxilla. He was able to open his mouth to the extent of half an inch only. Several of the teeth were loose, and pus could be forced by slight pressure from around their sockets.

In regard to his general condition, the patient seemed to be suffering from extreme debility; he could hardly arise from a sitting to a standing position without assistance, and walked with difficulty.

On inquiring into his previous history, it was found that he had been employed in (Hyatt's) a lucifer-match factory, on the corner of Broadway and one of the upper streets,

(36th,) for a number of months—that his health was excellent when he commenced work in this establishment, but had gradually failed; and that six weeks previously, the soreness and swelling had first made their appearance in the jaw. He stated, voluntarily, that several other persons employed in the manufactory were suffering from complaints similar to his. The fetor and difficulty of utterance in this case, together with the low grade of intelligence of the patient, prevented his attendants from getting as thorough a history of his case as they desired.

In view of the recent character of the disease of the jaw, and the bad general condition of the patient, he was ordered cod-liver oil, with iron, and an appropriate mouth-wash; several of the loose teeth were also removed, and a sympathetic abscess, which had formed below the jaw, was opened. Before the limits of the disease could be ascertained, with a view to relief by surgical means, the patient was removed from the hospital, on the 29th of March—the only changes in his condition comprising an improved state of his general health, and local relief, mainly in consequence of a new outlet for the discharge.

*Case* 3.—Catharine Karker, aged 21, born in Germany; single, poor, and moving in the lower ranks of life. Her occupation is that of filling match-boxes, at which she has been occupied since nine years of age; the disease began in the old factory, which was very badly ventilated. The whole lower jaw is involved. The disease began at the second molar tooth; she had a tooth extracted, and went back to work the same morning. The tooth was but decayed; and she had it extracted because it pained her. There was no disease previous to losing her teeth. She was under treatment by Dr. Ware, who removed pieces of bone several times.

*Case* 4.—Elizabeth Karker, aged 25, born in Germany; single, sister of the above. She is occupied in filling frames, and has been thus engaged twelve years. The lower jaw is involved; the disease has extended throughout the whole jaw. She says that the bone was removed to the middle of the chin at one operation. It commenced from having a tooth pulled; the dentist tore up a long strip of flesh, about the length of the index finger, attached to a piece of the bone. She entered the factory the next day; and from this date the disease commenced. A part of the stump is left. She had no disease previous to losing the tooth. She is under the treatment of Dr. Ware. An incision has been made at the articulation in a crucial form; the parts appear much deformed. This patient is now well, and pursues her avocation at the same factory, while her sister is still sick.

*Case* 5.—Catharine Brivogel, German; poor, single; aged 19 when disease commenced; it lasted for three years. Her occupation was that of dipper, which she followed nineteen months—during all this time her mouth was sore; she left at the end of nineteen months, and returned again in six months; ventilation of the factory was bad; in her case both jaws are involved; about half of left side of upper, and nearly same on lower, of the right side. The disease began in the upper jaw, about the first molar; she removed, herself, about half of the upper jaw, with the floor of the antrum still *in situ*—this piece she still keeps and exhibits; it has one tooth, the last molar, still remaining. She exhibits, also, thirteen teeth which she had extracted, and which were otherwise perfectly sound. The disease commenced from fracture of the jaw, while having a tooth extracted; there was pain in the jaw, but no disease previous to losing her teeth. She has been under the treatment of various physicians, among whom was Dr. Ware. She is now well, and has four teeth in the remaining portion of the lower jaw.

*Case* 6.—Mrs. Hellman, German; aged 25; when between seventeen and eighteen years of age, the disease commenced, and lasted eighteen months. She was engaged in filling boxes from the time she was a little girl. Her lower jaw was involved upon the left side, from the first canine tooth to the last molar. The disease began from a fracture of the jaw, while having a tooth extracted—she had it removed because it was crowded; she then caught cold, and inflammation occurred, followed by a discharge of pus. She had no toothache or pain in the jaw. There was no disease previous to having the tooth extracted. She was under the treatment of Dr. Ware, who removed the piece of bone.

These four cases were all from the same factory.

*Case* 7.—Julia Hatter, aged 20, German; single, poor; occupied as dipper for two years; lower jaw involved; left side first invaded, extending from first canine tooth backward; there is a fistulous opening through the skin, and free suppuration. She had a decayed tooth extracted for toothache; had no disease previous to having tooth drawn. This case is still going on, and is from a different factory.

The following cases are from the same factory as the patient whose case is given at length above:

*Case* 8.—Charles Jacobs, aged 27, German by birth, unmarried; resident in this country seven years; has been engaged in a lucifer-match factory five and a half years. The particular branch of the business in which he was employed was making the paste, on account of which he was much exposed to the vapor of phosphorus.

His disease commenced, about four years ago, with a simple toothache. This tooth, the last molar but one, was not decayed; but in the attempt to extract it, the crown was broken off. He returned directly to his work, without waiting for the wound to heal. The pain in the jaw did not cease, but gradually increased in severity. Suppuration was soon after established, and small fragments of bone were discharged. Necrosis of a large portion of the left side finally took place, and a considerable part of the jaw, from the symphysis to the articulation, was removed by a physician. Improvement of the general health followed this operation, and the parts cicatrized perfectly. The disease, however, still continued to extend upon the opposite side, involving new portions of the jaw in necrosis.

His general health continued very good; and he was able to pursue his work, with but occasional interruptions.

At the present time, the remainder of the jaw seems to be involved. The discharge of offensive matter is very great; loose sequestra can be felt along the track of the jaw; the patient's general health is failing, and there is evidence that, if the diseased bone is not removed, the case may terminate fatally before exfoliation is complete.

*Case* 9.—Amelia Miller, aged 21, born in Germany; has resided in this country eight years. She commenced work in the factory two years since, when nineteen years of age, and was then enjoying robust health. She was employed in cutting the matches and filling boxes. The disease commenced with a toothache. She applied to a dentist to have the tooth extracted, and in the attempt the tooth was broken off with a portion of the alveolar process. Her face swelled considerably, but she returned immediately to her work.

From this time, the disease seems to have gradually become developed, the pain grew more severe; suppuration was established, and matter was freely discharged from the gum, by the side of the teeth. Dead bone finally made its appearance in the diseased jaw, and large sequestra were removed from time to time. In this manner, the whole maxillary bone on the right side, extending from the symphysis to the angle, has been removed, and there remains a very firm cicatrix, covering a hard cartilaginous or bony rim, occupying the original position of the bone. All the teeth on the right side of the lower jaw are gone; but all motions of this part are well preserved.

The disease, however, is not arrested; upon the left side it is still extending, and gradually involving the healthy periosteum, and inducing necrosis of the remaining portion of the jaw. Her general health, which improved after the removal of the diseased mass, is now very good, but is, evidently, yielding to the renewed drain upon her system, and the constant irritation which she suffers. Unless the entire diseased bone be removed, there seems little hope that the disease will be arrested, short of complete destruction of the lower jaw.—*N. Y. Jour. Med.*

